# STAT3 inhibitor WP1066 as a novel therapeutic agent for renal cell carcinoma

**DOI:** 10.1038/sj.bjc.6605691

**Published:** 2010-05-11

**Authors:** A Horiguchi, T Asano, K Kuroda, A Sato, J Asakuma, K Ito, M Hayakawa, M Sumitomo, T Asano

**Affiliations:** 1 Department of Urology, National Defense Medical College, 3-2 Namiki, Tokorozawa-City, Saitama 359-8513, Japan

**Keywords:** STAT3, renal cell carcinoma, angiogenesis, apoptosis

## Abstract

**Background:**

Signal transducer and activator of transcription 3 (STAT3) regulates the expression of genes that mediate cell survival, proliferation, and angiogenesis and is aberrantly activated in various types of malignancies, including renal cell carcinoma (RCC). We examined whether it could be a novel therapeutic target for RCC by using the STAT3 inhibitor WP1066.

**Methods:**

The antitumour activities and related mechanisms of WP1066 were investigated *in vitro* on renal cancer cell lines and *in vivo* on murine xenografts.

**Results:**

In Caki-1 and 786-O renal cancer cells, 5 *μ*M WP1066 prevented the phosphorylation of STAT3, and 2.5 *μ*M WP1066 significantly (*P*<0.01) inhibited cell survival and proliferation. WP1066 suppressed the expression of Bcl-2, induced apoptosis, and inhibited the basal and hypoxia-induced expression of HIF1*α* and HIF2*α*, as well as vascular endothelial growth factor secretion into cell culture medium. Human umbilical vascular endothelial cells cocultured with media from WP1066-treated cells showed significantly reduced tubulogenesis (*P*<0.05). Systemic oral administration of WP1066 to mice for 19 days significantly inhibited the growth of Caki-1 xenograft tumours (*P*<0.05), and pathological analysis of xenografts of WP1066-treated mice showed decreased immunostaining of phosphorylated STAT3 and reduced length of CD34-positive vessels (*P*<0.05).

**Conclusion:**

Our results suggest that using WP1066 to inhibit the STAT3 signalling pathway could be a novel therapeutic strategy against RCC.

Signal transducer and activator of transcription (STAT) proteins have been shown to have important roles in cytokine and growth factor signalling pathways (Schindler and Darnell, 1995). These proteins are latent in cytoplasm and become activated through tyrosine phosphorylation, which typically occurs through cytokine-receptor-associated kinases, Janus kinases, or growth factor receptor tyrosine kinases (Schindler and Darnell, 1995). The activated STAT proteins then translocate to the nucleus and activate the transcription of target genes (Darnell, 1997). One of the STAT proteins, STAT3, was originally identified as an acute phase response factor activated by the interleukin-6 (IL-6) family of cytokines (Akira *et al*, 1994). Further studies have shown STAT3 to be activated in response to a variety of other cytokines and growth factors, including epidermal growth factor, platelet-derived growth factor, and transforming growth factor-*α*, as well as by oncogenic proteins such as Src and Ras (Schindler and Darnell, 1995; Darnell, 1997; Aggarwal *et al*, 2009). STAT3 has also been reported to regulate the transcription of key components of cell-cycle control and to modulate the activity of proteins regulating apoptosis (Zushi *et al*, 1998; Catlett-Falcone *et al*, 1999; Amin *et al*, 2004).

Signal transducer and activator of transcription-3 has been reported to be constitutively activated in various types of malignant tumours, and increased STAT3 activation has been recognised as an indicator of poor prognosis (Huang, 2007; Al Zaid Siddiquee and Turkson, 2008; Aggarwal *et al*, 2009). Constitutively activated STAT3 signalling in tumours has been shown to promote cell-cycle progression and prevent apoptosis by modulating cell cycle-associated and apoptosis-associated proteins such as cyclin D1 and Bcl-2; this is a possible mechanism of resistance to conventional chemotherapy and radiotherapy (Huang, 2007; Al Zaid Siddiquee and Turkson, 2008; Aggarwal *et al*, 2009). The activation of STAT3 has also been shown to upregulate vascular endothelial growth factor (VEGF) expression and promote tumour angiogenesis (Niu *et al*, 2002; Wei *et al*, 2003). STAT3 has therefore been considered as a potential therapeutic target for cancer treatment.

Renal cell carcinoma (RCC) is the most common malignant tumour of the adult kidney and accounts for approximately 3% of all adult cancer cases (Bilim *et al*, 2009; Thompson Coon *et al*, 2009). Unlike many other malignancies, RCC is generally resistant to conventional therapy (Bilim *et al*, 2009; Thompson Coon *et al*, 2009). Having previously reported a high frequency of increased STAT3 activation in RCC, especially in metastatic disease (Horiguchi *et al*, 2002b), we thought that STAT3 could be a novel therapeutic target for RCC and, in this study, examined whether the STAT3 inhibitor WP1066 shows antitumour activity against RCC *in vitro* and *in vivo*.

## Materials and Methods

### Cell culture and reagents

Human renal cancer cell lines Caki-1 and 786-O (American Tissue Culture Collection, Manassas, VA, USA) were used in this study, and WP1066 (Calbiochem, La Jolla, CA, USA) was dissolved in DMSO at 50 mM. The antibodies used in this study were antibodies against phospho-STAT3 (p-STAT3, Cell Signaling Technology Inc., Beverly, MA, USA), STAT3 (Cell Signaling Technology Inc), phospho-extracellular-activated protein kinase (ERK) (p-ERK, Cell Signaling Technology Inc.), ERK (Cell Signaling Technology Inc.), cleaved poly(ADP-ribose) polymerase (PARP) (Cell Signaling Technology Inc.), Bcl-xl (Cell Signaling Technology Inc.), Bcl-2 (Cell Signaling Technology Inc.), HIF1*α* (BD Transduction Laboratories, Lexington, KY, USA), HIF2*α* (Novus Biologicals, Littleton, CO, USA), and *β*-actin (Chemicon International Inc., Temecula, CA, USA). Horseradish-peroxidase-conjugated secondary antibodies and an enhanced chemiluminescence system were obtained from Amersham Pharm Biotech (Piscataway, NJ, USA).

### Cell viability assays and cell counts

Cell viability was assessed by the MTS assay using the Cell Titer96 AQueous Non-Radioactive Cell Proliferation Assay (Promega, Madison, WI, USA) according to the manufacturer's instructions. Briefly, cells were incubated overnight in 96-well plates (3 × 10^3^ cells/well) and then treated for 48 h with the indicated concentration of WP1066 (in DMSO) or with the corresponding amount of DMSO. Two hours after adding MTS, plates were read in a microplate autoreader at a wavelength of 490 nm. The results were expressed as the mean optical density of the six-well set for each group, and the plates were measured twice with similar results. For cell counts, after being incubated overnight in six-well plates (2 × 10^4^ cells/well in triplicate), cells were treated with 2.5 or 5 *μ*M WP1066 or with the corresponding amount of DMSO. Total cell numbers in three independent wells in each group were counted at the indicated time by using a hemocytometer, and the mean value of four fields was recorded.

### Determination of apoptosis

Apoptotic cells were assessed by double staining with FITC-conjugated annexin-V and propidium iodide. This staining was carried out using the Annexin V Apoptosis Detection Kit (Santa-Cruz Biotechnology Inc., Santa Cruz, CA, USA). To observe nuclear morphological changes with a fluorescence microscope, we fixed cells with 4% paraformaldehyde for 30 min at room temperature and stained them with 1 mM Hoechst 33258 (Sigma, St Louis, MO, USA).

### Western blot analysis

Cells that had been treated with 2.5 or 5 *μ*M WP1066 or the corresponding amount of DMSO for 24 h were lysed in RIPA buffer (10 mM Tris-HCl, 150 mM NaCl, 1% Triton X-100, 5 mM EDTA, 1% sodium deoxycholate, 0.1% SDS, 1.2% aprotinin, 5 *μ*M leupeptin, 4 *μ*M antipain, 1 mM phenylmethylsulphonyl fluoride, and 0.1 mM Na_3_VO_4_) before equal amounts of proteins were subjected to western blotting as described previously (Horiguchi *et al*, 2004).

### Hypoxia treatment and determination of VEGF production

Cells were plated at 1 × 10^4^ cells/well in six-well plates in RPMI 1640 containing 10% FCS, cultured for 24 h and then washed with serum-free medium and cultured for an additional 24 h in 1 ml of serum-free medium in the presence or absence of 5 *μ*M WP1066 under normoxic or hypoxic (1% O_2_) conditions. The medium was collected and centrifuged. The levels of VEGF in the supernatants were analysed using a Quantikine Human VEGF Immunoassay kit (R&D Systems, Minneapolis, MN, USA) and were normalised to the total protein content measured by the Bradford assay.

### Assessment of *in vitro* angiogenesis

*In vitro* angiogenesis was assessed according to the formation of capillary-like structures of human umbilical vascular endothelial cells (HUVECs) cocultured with human diploid fibroblasts as described previously (Horiguchi *et al*, 2004). The experimental procedure followed the instructions provided with the angiogenesis kit we used (Kurabo, Tokyo, Japan). Briefly, HUVECs were incubated in the conditioned medium, which was replaced at days 4, 7, and 9. On day 11, cells were fixed and stained using an anti-human CD31 antibody (Kurabo) according to the provided instructions. The formation of the capillary network was observed with a microscope at × 40 magnification. The total length of the tube-like structures in five randomly chosen microscope fields was measured by ImageJ (Bethesda, MD, USA) software.

### Xenograft model

Caki-1 cells (3 × 10^6^ cells per 100 *μ*l) were inoculated subcutaneously into the flanks of nude mice. When the average tumour diameter reached 5 mm, the mice were randomly divided into two groups (eight each), one of which was treated with WP1066 (40 mg kg^−1^) injected by oral gavage in a mixture of 20 parts DMSO to 80 parts polyethylene glycol 300 (Sigma-Aldrich, Tokyo, Japan) once per day (5 days on and 2 days off) as described elsewhere (Kong *et al*, 2008), and the other of which was treated with vehicle. The size of each tumour was monitored every 3 or 4 days until the 19th day after the first treatment. Tumour volume (mm^3^) was estimated using the following formula: volume=width^2^ × length × 0.52. Animal experimentation procedures were reviewed and approved by the National Defense Medical College's Animal Care and Use Committee.

### Immunohistochemistry

Immunohistochemistry was performed as described previously (Horiguchi *et al*, 2002b). Briefly, formalin-fixed, paraffin-embedded tumour sections (4 *μ*m) were deparaffinised in xylene and rehydrated through graded ethanols. The slides were placed in DAKO Target Retrieval Solution High pH (Dako Corporation, Carpinteria, CA, USA) and heated for 40 min for antigen retrieval. Endogenous peroxidase activity was quenched with Dako Peroxidase Blocking Reagent (Dako Corporation) for 10 min. The sections were incubated in 10% normal goat serum in PBS for 60 min at room temperature. After the sections were incubated overnight at 4°C with anti-phospho-STAT3 antibody, anti-STAT3 antibody, or anti-CD34 antibody (clone MEC14.7, Hycult Biotechnology, Uden, The Netherlands) at 1 : 50 dilution in PBS, the slides were stained using a Simple Stain Max PO kit (Nichirei Corporation, Tokyo, Japan) according to the manufacturer's instructions. Reaction products were visualised by immersing slides in DAB for 5 min. The vascularity in each tumour specimen was estimated using an anti-CD34 antibody. Three areas of most intense vascularisation were selected under a light microscope with a 100-fold magnification. The total length of the tube-like structures was measured by ImageJ software, and for each section, the mean length in the selected areas was recorded.

### Statistical analysis

The statistical significance of differences was evaluated by unpaired *t*-testing, and *P*-values less than 0.05 were considered to indicate significance.

## Results

### WP1066 inactivates STAT3 and suppresses renal cancer cell viability and proliferation

**Figure 1:**
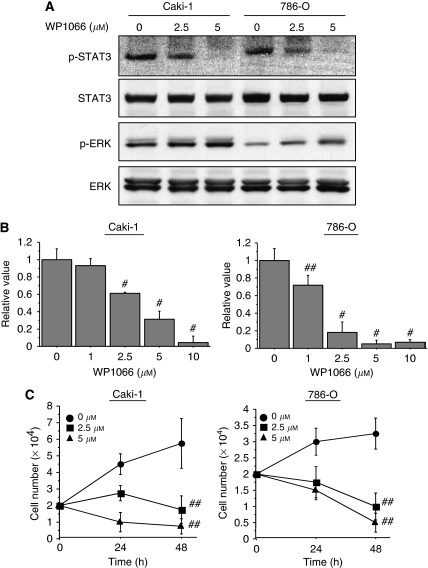
WP1066 inactivates STAT3 and suppresses the growth and viability of renal cancer cells. (**A**) WP1066 inhibited STAT3 phosphorylation, while stimulating ERK, in Caki-1 and 786-O cells. Caki-1 and 786-O cells were treated with the indicated concentration of WP1066 for 24 h and cell lysates were subjected to western blotting. (**B**) MTS-assay-determined viability of Caki-1 and 786-O cells treated with the indicated concentration of WP1066 for 24 h. Note the significantly lower viability of WP1066-treated cells (^#^, *P*<0.01; ^##^, *P*<0.05). The results are expressed as the mean ± s.e. of the three sets for each group. (**C**) Proliferation of Caki-1 and 786-O cells in six-well plates treated with the indicated concentration of WP1066. Total cell numbers were counted at 24 and 48 h. Note the significantly reduced proliferation of WP1066-treated cells (^##^, *P*<0.01). The results are expressed as the mean ± s.e. of the three sets for each group.

We first tested whether WP1066 inactivated STAT3 in Caki-1 and 786-O renal cancer cells. STAT3 is latent in the cytoplasm and its activation is accompanied by tyrosine phosphorylation at Tyr705, which induces dimerisation, nuclear translocation, and binding to DNA (Schindler and Darnell, 1995). Cell lysates were subjected to western blotting after Caki-1 and 786-O cells were incubated for 24 h with 2.5 or 5 *μ*M WP1066, concentrations known to suppress STAT3 activity (Ferrajoli *et al*, 2007; Iwamaru *et al*, 2007; Kong *et al*, 2008; Verstovsek *et al*, 2008). WP1066 inhibited phosphorylation of STAT3 in a dose-dependent manner (Figure 1A). We also examined its effect on another key component of signal transduction pathways and found that in both cell lines it stimulated the phosphorylation of ERK (Figure 1A).

We next examined the ways in which the viability and proliferation of renal cancer cells were affected when STAT3 activity was inhibited by WP1066. We treated Caki-1 and 786-O cells with the indicated concentration of WP1066 for 48 h and examined cell viability by MTS assay. The viability of both cell lines was significantly decreased in a dose-dependent manner (*P*<0.01) (Figure 1B). In triplicate observations made 1 and 2 days after Caki-1 and 786-O cells were treated with 5 *μ*M WP1066, it was found that the total number of WP1066-treated cells was significantly (*P*<0.01) less than the number of DMSO-treated cells (Figure 1C).

### WP1066 induces apoptosis in renal cancer cells

**Figure 2:**
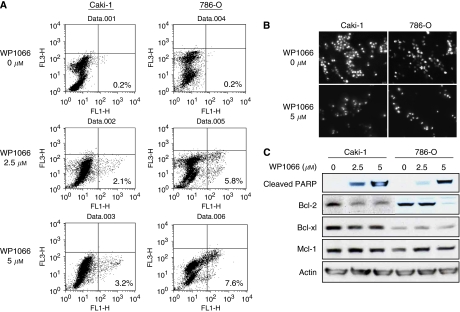
WP1066 downregulates antiapoptotic protein Bcl-2 and induces apoptosis in renal cancer cells. (**A**) Annexin V staining for apoptosis assay in Caki-1 and 786-O cells incubated for 24 h with the indicated concentration of WP1066. The percentages of annexin-V-positive- and propidium-iodide-negative (early apoptosis) fractions are indicated. (**B**) Nuclear morphologies observed by fluorescence microscopy after staining with Hoechst 33258 after Caki-1 and 786-O cells had been incubated for 24 h with the indicated concentration of WP1066. Note the fragmented and condensed nuclei characteristic of apoptosis. (**C**) Western blot analysis of apoptosis-related proteins. Cells were treated with the indicated concentration of WP1066 for 24 h and subjected to western blotting. Actin was used as an internal control.

Activated STAT3 has been shown to inhibit apoptosis by upregulating apoptotic regulatory proteins such as antiapoptotic proteins Bcl-2, Bcl-xl, and Mcl-1 (Zushi *et al*, 1998; Catlett-Falcone *et al*, 1999; Amin *et al*, 2004). To examine whether WP1066 would induce apoptosis in renal cancer cells, Caki-1 and 786-O cells incubated for 24 h with 2.5 or 5 *μ*M WP1066 were double stained with annexin-V and propidium iodide and were analysed by flow cytometry. The percentage of the population that was annexin positive and propidium iodide negative, which are apoptotic cells, was greater in cells treated with WP1066 than in control cells (Figure 2A). The nuclei of WP1066-treated cells seemed lobated and severely fragmented and contained highly condensed chromatin (Figure 2B), which are findings typical of apoptosis. To confirm apoptosis, we examined cells for the presence of PARP cleavage after treatment with WP1066. Cleaved PARP was evident in both Caki-1 and 786-O cells treated with WP1066 for 24 h (Figure 2C). The expression of Bcl-2 in both cell lines decreased 24 h after treatment with WP1066, whereas that of Bcl-xl or Mcl-1 did not (Figure 2C).

### WP1066 suppresses HIF1*α* and HIF2*α* expression and VEGF production

**Figure 3:**
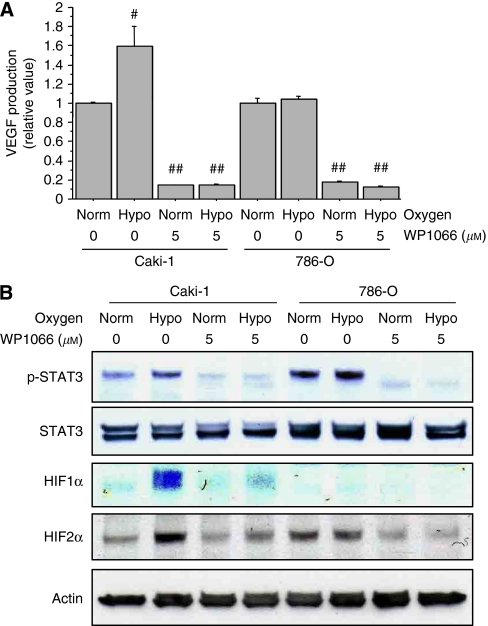
WP1066 downregulates HIF1*α* and HIF2*α* expression and reduces VEGF production in renal cancer cells. (**A**) Caki-1 and 786-O cells were incubated with the indicated concentration of WP1066 under normoxic (norm) or hypoxic (hypo, 1% O_2_) conditions for 24 h, and the VEGF levels in the cell culture media were measured by ELISA. Hypoxic conditions stimulated VEGF production in Caki-1 cells but not in 786-O cells (^#^, *P*<0.05). VEGF production under both normoxic and hypoxic conditions was significantly suppressed by treatment with WP1066 (^##^, *P*<0.01). The results are expressed as the mean ± s.e. of the three sets for each group. (**B**) Caki-1 and 786-O cells were incubated with the indicated concentration of WP1066 under normoxic or hypoxic (1% O_2_) conditions for 6 h, and cell lysates were subjected to western blotting. Hypoxic conditions stimulated STAT3 phosphorylation and upregulated HIF1*α* and HIF2*α* expression in Caki-1 cells, and these effects were suppressed by treatment with WP1066. Hypoxic conditions had no effect on STAT3 phosphorylation or HIF2*α* expression in 786-O cells, both of which were suppressed by treatment with WP1066.

Vascular endothelial growth factor is one of the most potent proangiogenic factors, and renal cancer cell lines, including Caki-1 and 786-O cells, have been shown to produce VEGF (Shinojima *et al*, 2007). Recent studies have identified that STAT3 is a direct transcription activator of the VEGF gene and that activation of STAT3 leads to tumour angiogenesis (Jung *et al*, 2005; Xu *et al*, 2005). We therefore examined whether WP1066 could suppress basal and hypoxia-stimulated production of VEGF. We incubated Caki-1 and 786-O cells with 5 *μ*M WP1066 under normoxic or hypoxic (1% O_2_) conditions for 24 h and measured VEGF levels in the supernatants. Caki-1 has the wild-type von-Hippel-Lindau (*VHL)* gene and expresses both HIF1*α* and HIF2*α*, whereas 786-O has a mutant *VHL* gene and expresses HIF2*α* but not HIF1*α* (Shinojima *et al*, 2007). It has been reported that HIF1*α* has a predominant role in VEGF production in Caki-1 cells but that HIF2*α* regulates VEGF production in 786-O cells (Shinojima *et al*, 2007). We found the VEGF level in Caki-1 cells incubated under hypoxic conditions to be significantly (60%) higher than that in Caki-1 cells incubated under normoxic conditions (*P*<0.05, Figure 3A). The VEGF level in 786-O cells, on the other hand, was not increased under hypoxic conditions (Figure 3A). WP1066 significantly (*P*<0.01) suppressed basal and hypoxia-stimulated VEGF production in both Caki-1 and 786-O cells (Figure 3A). A recent study has demonstrated that STAT3 increased the levels of HIF1*α* in Caki-1 cells by blocking its degradation and accelerating its *de novo* synthesis (Jung *et al*, 2005). We further examined whether WP1066 modulates HIF1*α* or HIF2*α* expression. In Caki-1 cells, hypoxic incubation increased the expression of HIF1*α*, HIF2*α*, and phosphorylated STAT3, and these increases were suppressed by WP1066 (Figure 3B). In 786-O cells, on the other hand, levels of HIF2*α* and phosphorylated STAT3 expression were not changed by hypoxic incubation but were suppressed by WP1066 (Figure 3B).

### WP1066 inhibits *in vitro* angiogenesis

**Figure 4:**
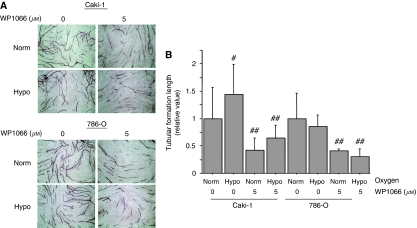
WP1066 inhibited *in vitro* angiogenesis. The HUVECs were incubated in a cell-conditioned medium with 5 *μ*M WP1066 under normoxic (norm) or hypoxic (hypo, 1% O_2_) conditions. After incubation for 11 days, HUVECs were fixed and stained with an anti-human CD31 antibody. (**A**) Representative photomicrographs ( × 40) of HUVEC tubular formations. (**B**) Tubular formation length was quantified in five randomly selected fields. (^#^, *P*<0.05; ^##^, *P*< 0.01 *vs* cells cultured without WP1066 under normoxic conditions). The results are expressed as the mean ± s.e. of the three sets for each group.

We next examined the effect of WP1066 on *in vitro* angiogenesis by using an HUVEC tubulogenesis assay. We incubated Caki-1 and 786-O cells with or without 5 *μ*M WP1066 under normoxic or hypoxic conditions for 24 h and collected the supernatants. These conditioned media were applied to HUVEC plates cultured in matrigel and the length of the tubular structures was measured. The tubular structures in HUVECs cultured in conditioned media obtained when Caki-1 cells had been incubated under hypoxic conditions were significantly (*P*<0.05) longer than those in HUVECs cultured in conditioned media obtained when Caki-1 cells had been incubated under normoxic conditions (Figures 4A and B). On the other hand, hypoxic incubation had no effect on the length of tubular structures in HUVECs cultured in conditioned media obtained from 786-O cells (Figures 4A and B). The length of tubular structures in HUVEC cultured in conditioned media obtained from both Caki-1 and 786-O cells treated with WP1066 was significantly (*P*<0.01) shorter than that in HUVECs cultured in conditioned media obtained from cells not treated with WP1066 (Figures 4A and B).

### WP1066 inhibits tumour growth in the murine xenograft model of Caki-1 cells

**Figure 5:**
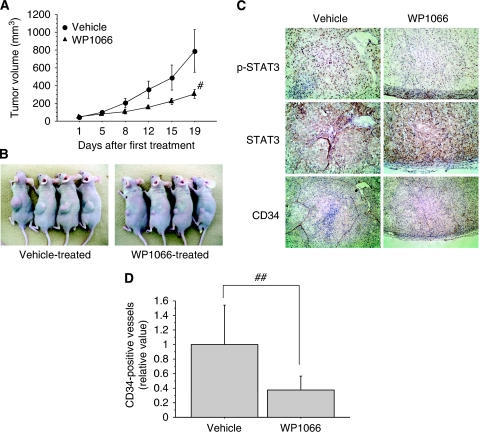
WP1066 inhibits tumour growth in the murine xenograft model of Caki-1 cells. (**A**) Established Caki-1 tumour xenografts in athymic male nude mice were treated with WP1066 (40 mg kg^−1^, *n*=8) or vehicle (20% DMSO-80% polyethylene glycol 300, *n*=8) by oral gavage once per day for 5 days (followed by a rest for 2 days) for a total of 19 days. Tumour volume in WP1066-treated mice on day 19 after the first administration was significantly smaller than that in control vehicle-treated mice (^#^, *P*<0.05). (**B**) Representative photographs of WP1066-treated mice and vehicle-treated control mice. (**C**) Immunohistochemical analysis of representative samples of xenograft tumours. Formalin-fixed xenograft tumours were immunostained with anti-p-STAT3, STAT3, or CD34 antibodies. Note decreased level of nuclear p-STAT3 immunostaining (upper right) and decreased tubular length of CD34-positive vessels (lower right) in WP1066-treated tumours compared with vehicle-treated control tumours. (**D**) Length of CD34-positive vessels was quantified in selected fields. (^##^, *P*<0.05).

As WP1066 inhibited the growth of renal cancer cells and angiogenesis *in vitro*, we tested the efficacy of WP1066 against renal cancer xenografts. WP1066 administrated orally is more efficacious and less toxic than WP1066 injected intraperitonealy or intravenously (Kong *et al*, 2008). When established Caki-1 xenografts were treated, 5 days on and 2 days off for a total of 19 days, with WP1066 (40 mg kg^−1^ orally) or only the vehicle (DMSO and polyethylene glycol 300), the mean volume of tumours treated with WP1066 (304.7±50.0 mm^3^) was significantly (*P*<0.05) smaller than that of tumours treated with only the vehicle (788.9±239.7 mm^3^, Figures 5A and B).

### WP1066 inactivates STAT3 *in vivo* and inhibits tumour angiogenesis

We next performed immunohistochemical analysis of Caki-1 xenograft tumours to examine whether WP1066 inhibited its growth by inactivating STAT3. STAT3 is latent in the cytoplasm and its activation is accompanied by tyrosine phosphorylation, which induces dimerisation, nuclear translocation, and binding to DNA (Schindler and Darnell, 1995). Consistent with the current understanding of STAT3 signalling pathways, predominant nuclear immunostaining of phosphorylated STAT3 was observed in the vehicle-treated control tumours (Figure 5C, upper left). In WP1066-treated tumours, on the other hand, there was little p-STAT3 immunostaining (Figure 5C, upper right). Similar total STAT3 immunostaining was observed in both vehicle-treated and WP1066-treated tumours, suggesting that WP1066 inhibited phosphorylation of STAT3 without modulating STAT3 expression (Figure 5C, middle row). To examine whether WP1066 inhibits tumour angiogenesis, we immunostained xenograft tumours with CD34 and measured the length of CD34-positive vessels in each tumour (Figure 5C, lower row). The mean total length of CD34-positive vessels in WP1066-treated tumours was significantly (*P*<0.05) shorter than that in vehicle-treated control tumours (Figure 5D).

## Discussion

In this study, we showed that in Caki-1 and 786-O renal cancer cells, STAT3 inhibitor WP1066 inactivated the STAT3 signalling pathway and induced apoptosis, accompanied by reduced Bcl-2 expression. WP1066 downregulated HIF1*α* and HIF2*α* expression under both normoxic and hypoxic conditions, resulting in reduced VEGF production and *in vitro* angiogenesis. Moreover, oral administration of WP1066 significantly suppressed tumour angiogenesis and inhibited the growth of xenograft tumours generated from Caki-1 cells. Our results suggest that inhibiting the STAT3 signalling pathway by using WP1066 could be a novel therapeutic strategy against RCC.

Activated STAT3 fosters tumourigenesis by preventing apoptosis, enhancing proliferation, angiogenesis, invasiveness, and immune evasion (Huang, 2007; Al Zaid Siddiquee and Turkson, 2008; Aggarwal *et al*, 2009). In various types of malignant tumours, including leukaemia and cancers of the breast, head and neck, melanoma, prostate, pancreas, and colon, activation of STAT3 is aberrantly increased (Huang, 2007; Al Zaid Siddiquee and Turkson, 2008; Aggarwal *et al*, 2009). We have previously identified a high frequency (50%) of increased STAT3 activation in RCC by immunohistochemical analysis (Horiguchi *et al*, 2002b). The STAT3 is also known to be involved in regulating cancer metastasis, and activation of STAT3 correlates with lymph node and distant metastasis (Huang, 2007). High levels of STAT3 activation are also significantly associated with the presence of metastasis and poor survival rate in RCC (Horiguchi *et al*, 2002b). These studies showed that inhibitors of the STAT3 signalling pathway have enormous potential in the treatment of cancer and drove us to examine its efficacy against RCC.

Several strategies for developing effective inhibitors of STAT3 have been pursued. One small-molecule STAT3 inhibitor, AG490, was originally selected from a group of tyrphostins screened for their ability to block Jak2 activity and has been shown to inhibit growth and induce apoptosis in some types of cancer cells, including renal cancer cells (Meydan *et al*, 1996; Horiguchi *et al*, 2002a). However, AG490 can inhibit STAT3 only at high concentrations (IC50 value=50–100 *μ*M) and therefore has not been shown to have an *in vivo* antitumour effect in animal models (Meydan *et al*, 1996; Horiguchi *et al*, 2002a). Priebe and his colleagues synthesised WP1066 by modifying the structure of AG490, and WP1066 has been shown to inhibit STAT3 activity and to have a potent antitumour effect on malignant glioma cells, both *in vitro* and *in vivo* (Iwamaru *et al*, 2007). Marked antitumour activity of WP1066 has also been shown against head and neck carcinoma, pancreatic cancer, bladder cancer, B-cell non-Hodgkin's lymphoma, and chronic myelogenous leukaemia (Ferrajoli *et al*, 2007; Kong *et al*, 2008; Verstovsek *et al*, 2008). We previously showed that the proliferation of Caki-1 cells is inhibited by high concentrations of AG490 (IC_50_ value=30.0 *μ*M) (Horiguchi *et al*, 2002a), and here we showed that the proliferation of Caki-1 cells is significantly inhibited by WP1066 at a concentration as low as 2.5 *μ*M. Moreover, oral administration of WP1066 significantly inhibited the growth of Caki-1 xenografts, and pathological examination showed reduced STAT3 activation in WP1066-treated xenografts. Thus, in accordance with previous studies (Ferrajoli *et al*, 2007; Hussain *et al*, 2007; Iwamaru *et al*, 2007; Kong *et al*, 2008; Verstovsek *et al*, 2008), WP1066 is a potent STAT3 inhibitor both *in vitro* and *in vivo*.

In addition to the inhibitory effect on STAT3 activation, WP1066 has also been shown to inhibit Jak2/STAT3 activation induced by cytokines and STAT5 (Ferrajoli *et al*, 2007; Iwamaru *et al*, 2007; Kong *et al*, 2008). In our preliminary experiments, we examined the activation of Jak2 and STAT5 in Caki-1 and 786-O cells cultured in media with 10% FBS and did not detect activated Jak2 or activated STAT5 in either cell line (data not shown). Moreover, treatment with WP1066 had no effect on their activation status. On the other hand, treatment of Caki-1 and 786-O cells with WP1066 simultaneously decreased STAT3 activation and increased ERK activation. The constitutive activation of ERK has been shown to occur frequently in RCC, especially in clear cell carcinoma, and the levels of activated ERK increased with increasing tumour size and tumour stage (Oka *et al*, 1995; Campbell *et al*, 2009). Moreover, increased activation of ERK is an independent indicator of poor prognosis in patients with RCC (Campbell *et al*, 2009). In our preliminary study, we further examined activated ERK expression immunohistochemically in xenograft tumours from mice treated with WP1066 and mice treated with vehicle. The staining intensity in tumours treated with WP1066 was much stronger than that in control tumours, which was the inverse of what was observed with p-STAT3 immunostaining (data not shown). Because ERK activation has been shown to be essential for mitogenic signals and to promote cancer proliferation and metastasis in various types of cancer including RCC (Campbell *et al*, 2009), WP1066-induced activation of ERK might compromise its antitumour activity.

Activated STAT3 has been shown to protect tumour cells from apoptosis by regulating genes encoding antiapoptosis-associated proteins, such as Bcl-2, Bcl-xl, and Mcl-1 (Zushi *et al*, 1998; Catlett-Falcone *et al*, 1999; Amin *et al*, 2004). Bcl-2 is frequently expressed in RCC, and a Bcl-2 overexpression may explain the resistance of RCC to conventional chemotherapy and radiotherapy (Tomita *et al*, 1996; Huang *et al*, 1999). We previously showed that AG490 inhibited STAT3 and induced apoptosis accompanied by the downregulation of Bcl-2 in renal cancer ACHN cells (Horiguchi *et al*, 2002a). In line with our previous study, we herein showed that WP1066 downregulated Bcl-2 expression effectively and induced apoptosis in both Caki-1 and 786-O cells.

Recent studies have identified STAT3 as a direct transcription activator of the *VEGF* gene and showed that activation of STAT3 leads to tumour angiogenesis *in vivo* (Niu *et al*, 2002; Wei *et al*, 2003). Mutations of VHL tumour suppressor protein are found in most clear-cell RCC, and loss of VHL protein function leads to elevated levels of HIF*α* and consequent overexpression of VEGF (Motzer *et al*, 2006). Recent studies demonstrated that STAT3 has a pivotal role in the regulation of HIF1*α* protein expression and stability and enhances HIF1*α*-mediated expression of VEGF by interacting with HIF1*α* (Jung *et al*, 2005; Xu *et al*, 2005). Here, we showed that incubating Caki-1 cells under hypoxic conditions activated STAT3, upregulated HIF1*α* expression, and enhanced VEGF production, and that all of these effects were inhibited by treatment with 5 *μ*M WP1066. Jung *et al* previously showed that AG490 inhibited hypoxia-induced activation of STAT3, as well as HIF1*α* expression and VEGF production, but this inhibition required a much higher concentration (30 *μ*M) (Jung *et al*, 2005). On the other hand, VEGF production is not stimulated by hypoxia in VHL-mutated 786-O cells (Shinojima *et al*, 2007). Accordingly, we found hypoxic conditions to have no effect on VEGF production or on STAT3 phosphorylation levels in 786-O cells, both of which were inhibited by treatment with WP1066. Interestingly, WP1066 markedly inhibited the expression of HIF2*α*, which has a crucial role in VEGF transcriptional regulation in 786-O cells (Shinojima *et al*, 2007). Our results suggest that not only HIF1*α* but also HIF2*α* might be regulated by STAT3. The HUVECs that were cocultured with the supernatants from Caki-1 and 786-O cells incubated with WP1066 showed reduced tubular formation, and our pathological assessment of the xenograft tumours showed that WP1066 reduced STAT3 activation and the length of CD34-positive microvessels. Our data suggest that WP1066 suppresses VEGF production and tumour angiogenesis under both normoxic and hypoxic conditions regardless of the *VHL* gene mutation status. To our knowledge, this report is the first to show that WP1066 inhibits tumour angiogenesis.

Surgical resection remains the mainstay of therapy for localised RCC, and metastatic RCC is highly refractory to conventional radiation therapy and chemotherapy (Bilim *et al*, 2009; Thompson Coon *et al*, 2009). The recent discovery and clinical development of some targeted agents have expanded treatment options in metastatic RCC (Escudier *et al*, 2009; Motzer *et al*, 2009), but complete response is rare and most patients eventually fail the treatment with targeted agents. The management of advanced RCC therefore remains a significant challenge to clinicians. Our study suggests that WP1066 is potentially useful for treating metastatic RCC.
